# Behavioral and quality-of-life outcomes in different service models for methadone maintenance treatment in Vietnam

**DOI:** 10.1186/s12954-016-0091-4

**Published:** 2016-02-02

**Authors:** Bach Xuan Tran, Long Hoang Nguyen, Vuong Minh Nong, Cuong Tat Nguyen, Huong Thu Thi Phan, Carl A. Latkin

**Affiliations:** Institute for Preventive Medicine and Public Health, Hanoi Medical University, Hanoi, Vietnam; Johns Hopkins Bloomberg School of Public Health, Baltimore, MD USA; School of Medicine and Pharmacy, Vietnam National University, Hanoi, Vietnam; Institute for Global Health Innovations, Duy Tan University, Da Nang, Vietnam; Authority of HIV/AIDS Control, Ministry of Health, Hanoi, Vietnam

## Abstract

**Background:**

Integrating HIV/AIDS and methadone maintenance treatment (MMT) services with existing health care delivery system is critical in sustaining efforts to fight HIV/AIDS in large injection-driven epidemics. However, efficiency of different integrative service models is unknown. This study assessed behavioral and health-related quality-of-life (HRQOL) outcomes of MMT in four service delivery models and explored factors associated with these outcomes of interest.

**Methods:**

A cross-sectional survey was conducted in two HIV epicenters in Vietnam: Hanoi and Nam Dinh Province. All patients in five selected MMT clinics were invited to participate, and 1016 were interviewed (80–90 % response rate).

**Results:**

Respondents had a mean age of 35.8, taken MMT for average 16.5 months and 3.3 % on MMT for 36–60 months. The MMT integrated with rural district health center (DHC) has the highest prevalence of concurrent drug use (11.3 %). The percentage of condom use (last sexual intercourse) with primary and casual partners was lowest in the MMT at urban DHCs. Patients at the rural DHC reported very high proportions of pain/discomfort (37.8 %), anxiety/depression (43.1 %), and mobility (13.3 %). In regression models, poorer HRQOL outcomes were found in MMT models in the rural areas or without general health care, and among those patients who were HIV positive, reported concurrent drug use, and had higher numbers of previous drug rehabilitation episodes. Mobility and anxiety/depression are factors that increased the likelihood of concurrent drug use among MMT patients.

**Conclusions:**

Outcomes of MMT were diverse across different integrative service models. Policies on rapid expansion of the MMT program in Vietnam should also emphasize on the integration with comprehensive health care services including psychological supports for patients.

## Background

In Asia, since injecting illicit drugs is recognized as a major risk factor for acquiring HIV, opioid substitution treatments have been considered an important component of HIV/AIDS prevention strategies [1–4]. Methadone maintenance therapy (MMT) has been widely used as a cost-effective intervention for opioid dependence. Evidences demonstrate the positive effects of MMT on people who use drugs (PWUD) by reducing the frequency of HIV-related behaviors and promoting health care access, health status, and HIV treatment outcomes [5–11]. Methadone has been included in the list of essential medicine for opioid management by the World Health Organization (WHO) in 2004 [12].

Along with HIV/AIDS, opioid illicit drug use was linked to other physical and psychological problems as well as high risk of mortality [6, 13–18]. Given the needs of PWUD for comprehensive medical care, the concept of integrating MMT with general health care services was proposed [9, 19–21]. It refers that various components of health services are provided by single or separate providers in one site. A wide range of literatures suggested the benefits of the integrating MMT to general health care facilities in accordance to clinical and public health perspectives. At patient level, this model facilitated health care utilization, improved health outcome, and treatment adherence [22–24]. At facility level, performing integrated services may avoid duplicating services and reduce administrative cost by utilizing fix components of facilities [25–29]. Besides, there are still several barriers that hamper the access and utilization of integrative clinics among drug users, including stigma and discrimination by health workers, acceptability of communities, the lack of comprehensive health care services, and the organization capacity for integration of different services [30–32].

In Vietnam, since the first MMT program was piloted in 2008, 156 MMT clinics have been established and operated with 28,000 DUs enrolling by April 2015 [33, 34]. With its large population of about 200,000 drug users, the Government of Vietnam has a strong political will and action plan to expand MMT services to cover 80,000 drug users by 2015 [7, 33, 35, 36]. Prior studies illustrated the influences of MMT on drug use behaviors, quality of life, and health care expenditure of HIV-positive PWUD [6, 8]. However, none of them took into account the impact of diverse MMT delivery models. In addition, the rapid cuts in foreign aids require Vietnam to identify strategies to reduce the deficit in resources for MMT as well as other HIV services and programs. Reducing costs, improving efficiency, and mobilizing resources from a wide variety of sources are potential policy options of which evidence on factors associated with the outcomes of service integration is necessary. The current organization of health service delivery system in Vietnam includes four levels: central, provincial, district, and commune [37]. Currently, MMT services are set up as a stand-alone site or integrated with provincial AIDS center (PAC), district health center (DHC), or regional polyclinics (RPC) which is a district-level health facility providing primary and secondary health care services for multiple communes far from the DHC [37]. The purposes of this study were to examine behavioral and health-related quality-of-life (HRQOL) outcomes of MMT in different service delivery models and explore the factors associated with these outcomes of interest.

## Methods

### Study settings and sampling

From January to August 2013, a cross-sectional survey was conducted in two HIV epicenters in Vietnam: Hanoi and Nam Dinh, with 20,717 and 3685 HIV-positive reported cases, respectively. Five clinics were purposively selected in Hanoi and Nam Dinh Provinces. These settings were selected based on some criteria: (1) providing MMT services; (2) covering a wide range of health care levels such as provincial, regional, and district levels; and (3) having adequate patients for the study. These sites were classified into four delivery models:

1. MMT + HIV voluntary testing and counseling services (VCT) at Nam Dinh PAC;
2. MMT + rural DHC in Xuan Truong District,
3. MMT + urban DHC in Tu Liem and Long Bien districts, and
4. MMT + urban Ha Dong RPC.

Both rural and urban DHCs in this study provide MMT along with antiretroviral treatment (ART) and general health care. Meanwhile, the Ha Dong RPC only provides general health care. During the period of this study, those services were co-located in one site with different health workers. Eligible subjects were 18 years or above, participating or having demand to enroll into the program. Patients meeting the criteria and presenting at the clinic during the whole study period were invited; and if they agreed to participate, an informed consent was given to them for signature. A total of 1016 respondents were recruited in the study. Since patients in our sample were receiving MMT free-of-charge, they did not receive any extra incentive for answering the survey. The response rate was 80–90 % across different sites (Table 1).

**Table 1:** Characteristics of study sites

Level	Settings	Site name	Type of services	Sample size
Province	Nam Dinh City	Provincial AIDS center	MMT + VCT	270
District (rural)	Xuan Truong District	District health center	MMT + VCT + ART + GH	151
District (urban)	Tu Liem District	District health center	MMT + VCT + ART + GH	201
District (urban)	Long Bien District	District health center	MMT + VCT + ART + GH	184
District (urban)	Ha Dong District	Regional polyclinic	MMT + GH	210

### Measures and instruments

Socioeconomic status, high-risk behaviors, and HRQOL of respondents were collected by face-to-face interview using structured questionnaires. Behaviors of interest include current drug use and condom use (last sexual intercourse) with intimate, casual, and commercial sex partners that were self-reported by respondents. HRQOL was measured by using EuroQol - five dimensions - five levels (EQ-5D-5L) instrument. The descriptive system includes five domains: mobility, self-care, usual activities, pain/discomfort, and anxiety/depression with five levels of response: no problems, slight problems, moderate problems, severe problems, and extreme problems, giving 3125 health states with respective single indexes. To compute those indexes, the EQ-5D-5L value set of Thailand was used in the absence of such values for Vietnam [38]. Additionally, the EuroQol - Visual Analog Scale (EQ-VAS) assesses the self-rated health of respondents in a scale from 0 to 100 points, labeled “the best health you can imagine” and “the worst health you can imagine.” The Vietnamese version of EQ-5D-5L was translated and has been widely applied in HIV and drug use populations of Vietnam [8, 15, 33, 38–40]. EQ-5D-5L and EQ-VAS have been shown to perform good measurement properties and be responsive in monitoring the health status of HIV-affected patients groups in Vietnam [5, 15, 33, 38–40].

### Statistical analysis

ANOVA and chi-squared test were used to assess the difference of characteristics and behavioral and HRQOL outcomes between different MMT models. Multivariate linear regression and logistic regression were performed to determine the factors related to outcomes of interest. Predictors of outcomes included sociodemographic characteristics, history of drug use and rehabilitation, health status and HIV infection, current drug-related behaviors, and service delivery models. Backward stepwise selection strategy was used to reduce the models, with variables having *p* values of log-likelihood ratio test <0.1 included. Statistical significance was set at *p* value <0.05.

### Ethical approval

The research was approved by the Scientific Committee of the Authority of HIV/AIDS Control, Ministry of Health, Vietnam.

## Results

A total of 1016 patients enrolled into the study, 997 taking MMT daily for average 16.5 months; 19 other patients were not included in this analysis since they had registered but not yet taken methadone. There were 19.7 % of the patients taking MMT for over 24 months; and 3.3 % were treated for 36–60 months. The mean age of patients was 36.7 (SD = 7.6) years. Among those, only 1.28 % were female, 67.7 % lived with spouse or partners. The majority had less than high school education (55.3 %) and cult of ancestors (or ancestral veneration) (88.2 %). The percentage of participants reporting HIV-positive status was 8.1 %. Compared to other sites, the rural MMT clinics integrated with Xuan Truong DHC have a large proportion of patients who were Catholic (25.8 %) and who were manual workers or farmers (35.8 %) (Table 2).

**Table 2:** Sociodemographic characteristics of respondents

			MMT + VCT + ART + DGH									
	MMT + VCT		Rural		Urban		MMT + RPC			All		*p* value
	Mean	SD	Mean	SD	Mean	SD	Mean	SD		Mean	SD	
Age	36.8	7.3	36.8	8.0	36.4	7.9	37.0	7.5		35.8	7.5	0.83
	*N*	%	*N*	%	*N*	%	*N*	%		*N*	%	
Sex (male)	266	98.5	151	100.0	206	98.1	380	98.7		1003	98.7	0.44
Educational attainment												
Illiterate	4	1.5	1	0.7	4	1.9	8	2.1		17	1.7	<0.01
Elementary	21	7.8	29	19.2	27	12.9	42	10.9		119	11.7	
Secondary	103	38.2	87	57.6	86	41.0	150	39.0		426	41.9	
High	121	44.8	28	18.5	81	38.6	157	40.8		387	38.1	
Vocational	12	4.4	5	3.3	7	3.3	8	2.1		32	3.2	
University	9	3.3	1	0.7	5	2.4	20	5.2		35	3.4	
Marital status												
Single	101	37.4	29	19.2	47	22.4	74		19.2	251	24.7	<0.01
Live with spouse	148	54.8	116	76.8	147	70.0	274		71.2	685	67.4	
Live with partner	0	0.0	0	0.0	1	0.5	2		0.5	3	0.3	
Divorced	19	7.0	6	4.0	15	7.1	32		8.3	72	7.1	
Widow	2	0.7	0	0.0	0	0.0	3		0.8	5	0.5	
Religion												
Cult of ancestors	247	91.5	96	63.6	198	94.3	355		92.2	896	88.2	<0.01
Buddhism	13	4.8	16	10.6	10	4.8	20		5.2	59	5.8	
Catholic	10	3.7	39	25.8	2	1.0	5		1.3	56	5.5	
Protestant	0	0.0	0	0.0	0	0.0	5		1.3	5	0.5	
Employment												
Unemployed	76	28.2	25	16.6	53	25.2	105		27.3	259	25.5	<0.01
Self-employed	159	58.9	67	44.4	112	53.3	204		53.0	542	53.4	
White collars	5	1.9	1	0.7	5	2.4	11		2.9	22	2.2	
Workers, farmers	10	3.7	54	35.8	18	8.6	18		4.7	100	9.8	
Students	0	0.0	0	0.0	0	0.0	2		0.5	2	0.2	
Other jobs	20	7.4	4	2.7	22	10.5	45		11.7	91	9.0	

In Table 3, history of drug use and previous drug use rehabilitation are compared among patients of four service models. On average, patients used drug for the first time at the age of 24.1 (SD = 6.5), for 13.5 years, and had about five episodes of drug rehabilitation prior to the MMT. Patients at the rural MMT clinic started using drugs at earlier ages and experienced fewer times of drug rehabilitation than patients at other clinics. The location of rehabilitation included self-management at home (71.6 %), voluntary centers (47.8 %), and compulsory centers (27.2 %). As for the reason for relapse, craving and peer inducement were the two major causes as reported by half of the patients.

**Table 3:** History of drug use and rehabilitation

			MMT + VCT + ART + DGH								
	MMT + VCT		Rural		Urban		MMT + RPC		All		*p* value
	Mean	SD	Mean	SD	Mean	SD	Mean	SD	Mean	SD	
History of drug use											
Age first used drug	24.3	7.0	26.6	7.4	25.3	6.7	23.5	6.1	24.1	6.5	0.21
Time since first episode (year)	13.5	5.1	11.2	5.3	12.2	5.9	14.5	6.2	13.5	5.7	<0.01
Time since first drug injection (year)	10.9	4.4	7.9	3.9	9.2	4.6	11.0	5.5	10.9	4.9	0.02
Drug rehabilitation											
Number of episodes	5.7	7.2	3.4	3.0	4.2	5.2	5.1	6.9	4.8	6.2	0.02
	*N*	%	*N*	%	*N*	%	*N*	%	*N*	%	
Location of rehabilitation											
Home, self-managed	213	85.9	103	75.7	132	68.8	226	61.8	674	71.6	<0.01
Voluntary centers	108	43.6	50	36.8	97	50.5	195	53.3	450	47.8	<0.01
Compulsory centers	74	29.8	13	9.6	32	16.7	137	37.4	256	27.2	<0.01
Reason for relapse											
Boredom	91	36.7	31	22.8	80	41.7	162	44.3	364	38.6	<0.01
Peer inducement	115	46.4	67	49.3	106	55.2	189	51.6	477	50.6	0.30
Craving	107	43.2	62	45.6	96	50.0	168	45.9	433	46.0	0.56
Unemployment	11	4.4	3	2.2	6	3.1	29	7.9	49	5.2	0.02

Table 4 presents the current drug use, condom use, and self-reported HRQOL of patients participating in each MMT model. We found heterogeneity in these outcomes across different service models, but it was largely contributed by geographical location. There were less than 5 % of the patients who reported current drug use during MMT; however, it was the highest in the rural MMT integrated with Xuan Truong DHC (13.1 %). Among sexually active individuals, most services also, the percentage of patients using condom (last sexual intercourse) with their primary and casual partners was lowest in urban MMT at DHC in comparison with other clinics. Overall, 95.4 % of the patients reported that their HRQOL had improved to some extent over MMT. The proportion of patients who reported having any health problem across five dimensions of the EQ-5D instrument in the rural areas was double than that of all patients in the sample. Patients who were attending the urban MMT integrated with Ha Dong RPC reported smallest proportions of all health problems among selected sites.

**Table 4:** Behavioral and quality-of-life outcomes by different service models

			MMT + VCT + ART + DGH								
	MMT + VCT		Rural		Urban		MMT + RPC		All		*p* value
	*N*	%	*N*	%	*N*	%	*N*	%	*N*	%	
Concurrent drug use	15	5.6	17	11.3	11	5.2	6	1.6	49	4.8	<0.01
Condom use (last sexual intercourse)											
Intimate partners	58	30.4	40	30.5	30	17.4	97	31.7	225	28.1	0.01
Casual partners	6	75.0	11	64.7	4	30.8	14	60.9	35	57.4	0.15
Commercial sex partners	16	94.1	16	88.9	11	91.7	28	77.8	71	85.5	0.35
Improved QOL	255	94.8	132	97.1	205	97.6	360	94.0	952	95.4	0.18
Reported health problems											
Mobility	19	7.0	20	13.3	15	7.1	20	5.2	74	7.3	0.02
Self-care	10	3.7	9	6.0	9	4.3	12	3.1	40	3.9	0.49
Usual activities	18	6.7	17	11.3	9	4.3	16	4.2	60	5.9	0.01
Pain or discomfort	37	13.7	57	37.8	34	16.2	52	13.5	180	17.7	<0.01
Anxiety or depression	56	20.7	65	43.1	38	18.1	51	13.3	210	20.7	<0.01
	Mean	SD	Mean	SD	Mean	SD	Mean	SD	Mean	SD	Mean
Health utility											
EQ-5D single index	91.2	15.1	82.7	16.5	91.4	15.3	93.3	13.4	83.4	20.5	<0.01
VAS	76.4	15.3	75.8	16.2	78.8	14.3	78.3	13.0	74.8	16.8	0.09

Table 5 explored the related factors with current drug use and HRQOL of MMT patients in the reduced linear regression models. These models specified the association between service models and duration of MMT with the outcomes of interest while adjusting for other covariates. Although no significant difference in current drug use between service models was found, MMT clinics integrated with urban DHC and RPC showed better HRQOL outcome measured using VAS, and the rural MMT showed the lowest EQ-5D index score. Duration on MMT is also associated with reduced likelihood of current drug use among those retained on MMT, while having any problems in mobility (OR = 4.2) and anxiety/depression (OR = 3.1) during MMT substantially increased the risk of current drug use.

**Table 5:** Factors associated with concurrent drug use and quality-of-life outcomes of MMT

	EQ-5D index		VAS		Concurrent drug use	
	Coefficient	95 % CI	Coefficient	95 % CI	OR	95 % CI
MMT model (MMT + VCT - reference)						
Rural MMT-ART-VCT-DGH	−4.7**	(−8.7; −0.6)	1.4	(−2.1; 4.9)	0.39	(0.08; 1.94)
Urban MMT-ART-VCT-DGH	1.2	(−1.8; 4.1)	4.0***	(1.3; 6.7)	0.96	(0.30; 3.04)
MMT +regional polyclinic	−0.1	(−3.1; 2.9)	3.7***	(0.9; 6.4)	2.38	(0.90; 6.26)
Duration of MMT (years)	0.01	(−0.1; 0.1)	−0.1*	(−0.2; 0.0)	0.91***	(0.86; 0.96)
Reported HIV status						
Positive vs. negative			−4.6***	(−7.8; −1.5)		
Reported health problems (yes vs. no)						
Mobility					4.20**	(1.41; 12.51)
Anxiety or depression					3.09**	(1.27; 7.53)
Concurrent drug use						
Yes vs. no	−9.2***	(−14.7; −3.7)	−7.3***	(−12.3; −2.4)	N/A	
History of drug rehabilitation						
Number of episodes	−0.2**	(−0.4; −0.0)				
Education (illiterate - reference)						
Elementary	14.4***	(5.8; 22.9)				
Secondary	14.7***	(6.5; 22.9)				
High	14.7***	(6.5; 22.9)				
Vocational	13.6***	(3.6; 23.6)				
University	15.4***	(5.6; 25.3)				
Marital status (single - reference)						
Widow	−16.2*	(−32.8; 0.3)				
Religion (cult of ancestors - reference)						
Buddhism	−4.0*	(−8.8; 0.7)				
Catholic	−7.2***	(−12.5; −1.8)				
Employment (unemployed - reference)						
Self-employed	2.3**	(0.1; 4.4)	5.4***	(3.2; 7.7)		
White collars			11.6***	(3.2; 20.0)		
Workers, farmers			3.9*	(−0.1; 7.9)		
Other jobs			4.2**	(0.4; 8.0)		
Age group (18 to <25 - reference)						
25 to <30	6.9***	(3.0; 10.8)				
30 to <35	6.9***	(3.4; 10.4)	−2.8*	(−5.7; 0.1)	2.26*	(0.99; 5.18)
35 to <40	4.9***	(1.4; 8.4)	−3.9**	(−6.9; −0.9)		
40 to <45	7.0***	(3.0; 11.0)	−4.1**	(−7.5; −0.7)		
45 and above			−11.8***	(−15.4; −8.3)		
Constant	72.0***	(63.1; 80.9)	77.7***	(74.4; 81.0)		

CI in *parentheses*

***p < 0.01; **p < 0.05; *p < 0.1

There are several sociodemographic factors and history of drug use associated with HRQOL outcomes of MMT. Unemployment, HIV-positive status, concurrent drug use, and higher numbers of previous drug rehabilitation episodes were associated with decreased HRQOL among MMT patients.

## Discussion

While integration and decentralization of HIV/AIDS and substance abuse treatment services with existing health care delivery system is critical in sustaining efforts to fight HIV/AIDS in large injection-driven epidemics, findings of this study showed a significant heterogeneity in outcomes of MMT across different service models. However, variability in MMT outcomes was largely contributed by the geographical differences. Although previous studies determined that long-term MMT in general will bring about improvements in health status and reduce the likelihood of concurrent drug use among patients [41]; we found poorer HRQOL outcomes in rural or lower level MMT clinics. If the goal is to engage drug users with MMT in a timely manner and prevent HIV transmission, it is necessary that not only HIV-related interventions but also general health care should be provided [13, 35, 36, 42]. These findings support current policies on scaling up MMT program in Vietnam and inform the development of more comprehensive care and support services for drug users as well as building capacity of health workers in substance abuse treatment in large drug-using populations.

This is the first study profiling the outcomes of different integrative models for delivering MMT. It contributes to the literature empirical evidence that the integration of MMT with existing health care services will yield better outcomes [36, 43]. In literatures, integration has potential roles to facilitate the continuity of care and increased access to medical services [19, 44–46]. Besides, patients participating in this model have lower costs for health care due to fewer admissions to hospitals, reducing their health care expenditure and household economic burden [8, 33, 47]. Those reasons may results in better health outcomes of patients in integrated clinics at PRC and urban DHC compared to other clinics.

This study reaffirms the reduced drug use behaviors over the course of MMT that support previous study on the cost-effectiveness of short-term MMT for drug users regardless of their HIV status [5, 7]. The overall HRQOL score measured using EQ-5D in this study was higher than HIV-positive drug users taking MMT and lower than the general population in Vietnam [6, 8, 14, 15, 38, 39]. However, patients attending rural or decentralized services had clinically important differences in HRQOL compared to others. We observed a very high proportion of having problems in pain/discomfort and anxiety/depression and notably in mobility among patients attending MMT at the rural DHC. The proportion of having any problem in mobility of the general population and HIV-positive group was 2.1 and 7.5 %, respectively [15]. In measuring health-related quality of life, VAS is a valid measure that captures the values of patients attached to their current health states which is no different than the gold standard method for measuring preference-based HRQOL—the Standard Gamble [39]. VAS score had not increased over long-term MMT that could be explained by the fact that drug users receiving MMT still have many other social, economic, and health concerns [6, 13, 14, 38, 48]. As observed in previous studies in Vietnam, we found that HIV status, current drug use, history of drug rehabilitation, duration of drug use prior to MMT, and various socioeconomic characteristics of respondents were significantly associated with HRQOL outcomes [6, 8, 13–15, 38, 48–50].

Integration has been raised as a priority in the context of limited resources for HIV/AIDS responses [35, 36, 51]. In economic theory, this model has the potential advantages on technical (focusing on unit cost of services) and allocative efficiency (focusing on cost-effectiveness of services) [52]. Recent studies confirmed that integrated HIV/AIDS service delivery was more efficient than stand-alone services [9, 20]. However, none of them mentioned the efficiency of integrated MMT services with general/primary health care. Findings of this study have implications to inform the expansion and management of MMT services in Vietnam. First, the majority of patients registering at new MMT sites might be younger and have more physical and mental health problems. Depression and other mental disorders have been known as a predictor of drug relapse and HIV risk behaviors and negatively affect antiretroviral treatment outcomes [53–55]. Therefore, integrating MMT clinics with general health care facilities is necessary, and general health care and psychological support should be provided to drug users prior to and during MMT. Second, in the rural areas, the long distance to MMT clinics can be a barrier to the access and adherence of patients. It is important to notice that in this analysis, 13.3 % of the patients have problems in mobility, and this group is about four times more likely to use drugs concurrently than their counterpart. Since patients require daily uptake of the medication, a satellite model that links the MMT at DHC with commune health stations for delivering MMT in large drug-using populations could be highly efficient. Besides, take-home dose may also be an option that helps overcome the geographical barriers and improve adherence of patients. However, with the current policies, the management and delivery of methadone medication that does not support the implementation of take-home dose in short term is restricted.

The strength of this study was the participation of a large sample size in various levels of the health care system. In addition, validated instruments (EQ-5D-5L and VAS) were employed to allow for the comparability of measurements. However, the study has limitations. First, the cross-sectional design may not allow the causal relations between MMT delivery models and the changes of HRQOL as well as risk behaviors of respondents. Second, the collected data was based on self-reported information, which was subject to desirability bias due to respondents’ recall. In this study, concurrent drug use was self-reported that might underestimate the actual prevalence among MMT patients. In addition, we only interviewed the patients who remained at the MMT clinics while missing those who dropped out of the program. In addition, we did not have information regarding MMT doses and patient responses. Finally, the generalization of study was limited due to convenience sampling technique.

## Conclusions

In conclusion, the study supports the effectiveness of MMT for drug users in Vietnam and preferable outcomes of integrating MMT with existing health services. Heterogeneities in behavioral and HRQOL outcomes across different integrative MMT service models suggest a need not only to provide HIV-related interventions but also the value of comprehensive health care including psychological supports for MMT patients especially in rural areas. Future research should examine the costs and efficiency of satellite models for dispensing MMT where distance to the clinic is a barrier to service access and utilization.
